# Suramin inspires next-generation broad-spectrum antiviral design targeting RdRps

**DOI:** 10.1371/journal.ppat.1014766

**Published:** 2026-11-06

**Authors:** Arwen Baer, Ge Yang, Fan Bu, Bin Liu

**Affiliations:** 1 Section of Transcription & Gene Regulation, The Hormel Institute, University of Minnesota, Austin, Minnesota, United States of America; 2 Department of Chemistry, Gustavus Adolphus College, St Peter, Minnesota, United States of America; 3 Department of Pharmacology, University of Minnesota Medical School, Minneapolis, Minnesota, United States of America; Washington University School of Medicine in Saint Louis: Washington University in St Louis School of Medicine, UNITED STATES OF AMERICA

## Abstract

Structural studies of diverse viral RNA-dependent RNA polymerases reveal that the broad-spectrum antiviral Suramin binds distinct sites while repeatedly exploiting conserved RNA-interacting and electrostatic features. This Pearl highlights common vulnerabilities within viral RNA polymerases and discusses how structural studies provide a framework for future antiviral design.

Many medically important viruses store their genetic material as RNA and rely on RNA-dependent RNA polymerases (RdRps) for genome replication and transcription. RdRps share a conserved right-handed structure with fingers, palm and thumb subdomains connected by channels that play a major role in the entry of nucleotide triphosphates (NTPs) and template RNA into the active site [1]. This remarkable structural conservation has made viral polymerases attractive targets for broad-spectrum antiviral development. To date, however, most clinically approved RdRp inhibitors are nucleoside analogues that target the catalytic center. Although highly successful in some cases, these inhibitors remain susceptible to resistance mutations, require intracellular metabolic activation, and exploit only a limited portion of the polymerase surface. Meanwhile, non-nucleoside inhibitors such as ERDRP-0519 and AS-136A have broadened the chemical strategies available for targeting the RdRp active center [2]. Conserved non-catalytic RNA-binding channels therefore represent an underexplored opportunity for developing orthogonal antiviral mechanisms that complement catalytic-site inhibition while potentially reducing the impact of resistance.

## Suramin: A century-old drug with broad antiviral activity

Suramin is a century-old drug that remains in clinical use as an antiparasitic to treat sleeping sickness and river blindness. Since its synthesis in 1916, Suramin has been investigated for applications ranging from parasitic disease and viral infection to cancer, snakebite, and autism [3]. This wide range of functions reflects its highly polypharmacological nature. Structurally, Suramin consists of two trisulfonated naphthalene moieties connected by flexible linkers (Fig 1a), resulting in six negatively charged sulfonate groups that promote electrostatic interactions with positively charged protein surfaces and nucleic acid-binding interfaces.

**Fig 1:**
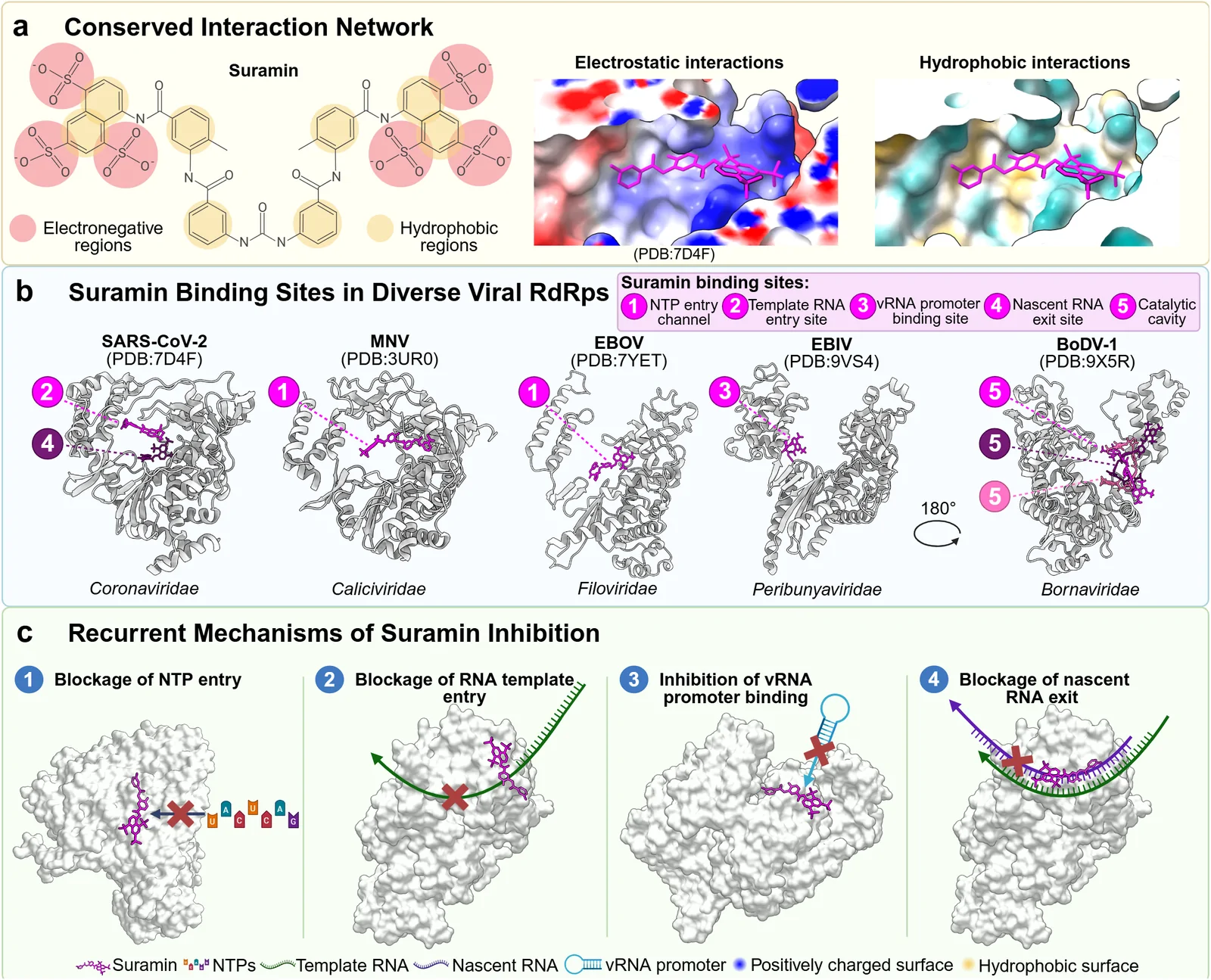
Mechanisms of Suramin-mediated inhibition across viral RNA polymerases. **(a)** Conserved interaction network. The negatively charged sulfonate groups of Suramin interact with electropositive RNA-binding environments, steering it in clefts where hydrophobic residues anchor it in place. These features are observed across diverse viral polymerases despite differences in structure. **(b)** Close-up views of Suramin bound within the catalytic chamber of the SARS-CoV-2 RdRp-Suramin, MNV RdRp-Suramin, EBOV RdRp-Suramin, and EBIV RdRp-Suramin complexes, as well as the Suramin-bound BoDV-1 L-P complex. **(c)** Recurring mechanisms of inhibition across viral polymerases. Suramin blocks the nucleotide entry channel, RNA template entry, viral promoter binding, and the exit path of nascent RNA. Figure was prepared using ChimeraX (https://www.cgl.ucsf.edu/chimerax/) and BioRender (https://biorender.com/20yqrs9).

Consistent with these properties, Suramin has displayed broad antiviral activity against many RNA and DNA viruses, including human immunodeficiency virus (HIV), dengue virus, Zika virus (ZIKV), and herpes simplex virus type 1 [4–7]. Despite this remarkable breadth of antiviral activity in biochemical and cell-based assays, Suramin has not been successfully translated into a clinically useful antiviral. Clinical development for HIV infection was discontinued because of dose-limiting toxicities and an unfavorable therapeutic index despite measurable antiviral activity. For most other aforementioned viruses, Suramin has remained largely confined to biochemical, cell-based, or limited preclinical animal studies without clinical translation. These limitations largely reflect its unfavorable physicochemical properties, including its high molecular weight (~1.3 kDa), extensive plasma protein binding, poor cellular permeability, and broad target promiscuity [3], all of which restrict intracellular target engagement.

Historically, Suramin has therefore been viewed primarily as an extracellular antiviral. Numerous studies have demonstrated inhibition of viral attachment, receptor engagement, and membrane fusion [3]. However, biochemical studies have also established that Suramin directly inhibits viral RNA synthesis, blocking RNA polymerization by murine norovirus (MNV) RdRp and primarily impairing RNA initiation in chikungunya virus [8,9]. More recent structural studies have provided a molecular basis for these activities by revealing how Suramin directly engages viral RdRps. Rather than acting exclusively at the cell surface, Suramin directly binds diverse viral RdRps by occupying conserved RNA-binding channels outside the catalytic center. The recurrence of this binding mode across evolutionarily distinct viral polymerases suggests that Suramin recognizes conserved functional regions rather than virus-specific binding pockets. These observations suggest that Suramin may represent more than a candidate for antiviral repurposing. Instead, it could serve as a privileged starting scaffold for the rational development of next-generation non-nucleoside inhibitors targeting conserved RNA-binding regions across viral polymerases.

## Suramin targets conserved RNA-binding landscapes

Despite substantial sequence divergence, viral RdRps utilize conserved electropositive channels to guide template RNA and NTPs into the catalytic center. Structural studies consistently show that Suramin occupies these RNA-associated pathways rather than the catalytic nucleotide-binding pocket itself (Fig 1b).

This strategy is most apparent in SARS-CoV-2, murine norovirus and Ebola virus (EBOV) polymerases, where Suramin binds within channels required for template RNA or NTP entry (Fig 1c). In SARS-CoV-2, Suramin simultaneously occupies the template entry, nascent RNA exit, and an adjacent NTP-access site, sterically preventing productive RNA synthesis [10]. In MNV and EBOV, Suramin binds the positively charged NTP channel, disrupting substrate positioning required for catalysis [8,11]. In EBOV, the bound Suramin molecule further extends toward the nascent RNA path, potentially interfering with RNA elongation [11]. Across other viral polymerases, Suramin inhibits RNA synthesis through related mechanisms at earlier stages. In Ebinur Lake virus (EBIV) and Crimean-Congo haemorrhagic fever virus (CCHFV), Suramin occupies positively charged promoter-binding regions required for recognition of the viral RNA promoter, thereby preventing replication initiation [12,13]. CCHFV uses a dual-site mechanism involving promoter recognition and an allosteric site on the Linker-Finger domain [13], whereas EBIV contains two distinct inhibitory sites that disrupt both promoter binding and RNA elongation [12]. In contrast, Borna disease virus 1 (BoDV-1) contains a single catalytic cavity occupied by three stacked Suramin molecules that directly occlude the active site [14].

Remarkably, although Suramin occupies different locations in different viral polymerases, its mode of recognition remains consistent. Across diverse RdRps, the negatively charged sulfonate groups engage electropositive RNA-binding environments enriched in lysine, arginine, and other polar residues, whereas the aromatic naphthalene rings participate in hydrophobic packing interactions that stabilize binding within the pocket (Fig 1a). This preference for electropositive nucleic-acid-binding surfaces may limit selectivity, as Suramin can also inhibit host DNA polymerases α and δ [15]. In SARS-CoV-2, Suramin is anchored within the template-binding cleft through a combination of electrostatic, hydrogen-bonding, and hydrophobic interactions [10]. A similar recognition strategy is observed in EBIV and CCHFV polymerases, where Suramin occupies positively charged promoter-binding regions [12,13]. In CCHFV, the sulfonate groups are proposed to mimic the phosphate backbone of the viral RNA promoter [13]. Comparable electrostatic, hydrogen-bonding, and hydrophobic interactions are also observed in EBOV and BoDV-1, where they stabilize Suramin within RNA-associated channels [11,14].

Collectively, these observations indicate that although the number and spatial arrangement of Suramin-binding sites differ among viral polymerases, Suramin consistently recognizes conserved electrostatic environments required for RNA binding rather than specific amino acid sequences. This shared recognition strategy provides a unifying structural explanation for its broad antiviral activity (Fig 1a).

## Implications for next-generation antiviral therapeutics

Despite broad antiviral activity, Suramin is unlikely to become an ideal therapeutic because of its high molecular weight, extensive plasma protein binding, poor cellular permeability, polypharmacology, and associated toxicities. Nevertheless, recent structural studies fundamentally change how Suramin should be viewed in antiviral drug discovery. Rather than representing another opportunity for drug repurposing, Suramin demonstrates that evolutionarily diverse viral polymerases converge on conserved electropositive RNA-binding environments that are essential for viral RNA synthesis. Unlike the catalytic center targeted by nucleoside analogues, these non-catalytic functional regions represent an underexplored class of antiviral targets that could complement existing therapies, expand the accessible chemical space for inhibitor design, and potentially reduce selective pressure associated with catalytic-site resistance.

## Future directions

Recent advances provide a strong foundation for translating these discoveries into antiviral therapeutics. Multiple Suramin derivatives retain antiviral activity, with some displaying greater potency than the parent compound [10]. Structural comparisons between Suramin and the derivative NF023 revealed similar binding modes within the MNV RdRp, suggesting that the conserved interaction network within electropositive RNA-binding channels can be preserved despite substantial scaffold modifications [8]. Likewise, derivatives containing fewer sulfonate groups have retained antiviral activity while displaying improved physicochemical properties, suggesting that the key pharmacophoric features responsible for polymerase recognition can be maintained while reducing molecular weight, improving membrane permeability, and enhancing target specificity [16]. Alternative delivery strategies, such as glycol chitosan-based nanoparticles, may further improve intracellular delivery and antiviral efficacy [10]. Accordingly, improved intracellular delivery should be coupled with scaffold optimization to enhance viral target selectivity and minimize host off-target effects. Together, these advances suggest that the antiviral activity of Suramin can be decoupled from many of its unfavorable pharmacological properties, providing a practical foundation for the development of next-generation non-nucleoside antivirals.

The growing availability of high-resolution polymerase structures now enables a transition from empirical screening to structure-guided antiviral design. Rather than optimizing Suramin itself, future optimization of Suramin could focus on truncating or modifying its poorly resolved arm, particularly the terminal naphthalene-trisulfonate group, as structural studies suggest unequal contributions of its two halves to polymerase binding [10,11]. The identification of distinct binding pockets with different contributions to affinity further supports the design of pocket-specific asymmetric analogues [12]. Selective reduction of sulfonate groups, together with optimization of the aromatic scaffold and central urea linker, may reduce nonspecific electrostatic interactions, improve target selectivity, and potentially enhance membrane permeability and blood-brain barrier penetration by decreasing molecular size and negative charge [3].

An important question is whether these conserved recognition principles extend beyond viral polymerases. Although Suramin inhibits both viral entry and replication in several RNA viruses, including ZIKV and enterovirus A71, high-resolution structures of Suramin bound to viral entry proteins remain unavailable [7,17,18]. Resolving these complexes will determine whether the conserved electrostatic recognition observed in RdRps also governs inhibition of viral attachment and membrane fusion. Beyond viral entry, Suramin also disrupts genomic RNA binding by the nucleocapsid proteins of Rift Valley fever virus and the SARS-CoV-2, extending this recognition principle to other viral proteins [19–21]. Among the viruses studied to date, EBOV may represent a particularly compelling system for evaluating this concept. Suramin is shown to inhibit both viral entry and the viral L–VP35 polymerase while suppressing viral RNA replication with a favorable *in vitro* selectivity index [11]. Whether these activities act synergistically remains unknown, but simultaneous inhibition of multiple stages of the viral life cycle may provide greater antiviral robustness than targeting either process alone. This possibility may be especially relevant for filoviruses, which produce prolonged systemic viremia with exceptionally high viral titers in the bloodstream, potentially increasing the contribution of extracellular inhibition while still requiring effective suppression of intracellular replication. As no small-molecule antiviral has yet achieved regulatory approval for Ebola virus disease, dual-mechanism inhibitors capable of targeting both viral entry and genome replication warrant further investigation. Such strategies, however, will require improved selectivity, as similar electropositive surfaces are also present in host nucleic-acid-binding proteins.

More broadly, these findings suggest that future inhibitor design should focus on conserved physicochemical environments, such as electropositive channels and complementary hydrophobic surfaces, rather than attempting to target identical binding pockets. Such conserved functional landscapes may represent a previously underappreciated class of antiviral targets and provide a conceptual framework for developing broad-spectrum therapeutics against emerging viral pathogens while reducing susceptibility to resistance associated with catalytic-site inhibitors.
